# Optimal treatment for spermatogenesis in male patients with hypogonadotropic hypogonadism

**DOI:** 10.1097/MD.0000000000016616

**Published:** 2019-08-02

**Authors:** Jianli Lin, Jiangfeng Mao, Xi Wang, Wanlu Ma, Ming Hao, Xueyan Wu

**Affiliations:** a Department of Endocrinology, Peking Union Medical College Hospital, Peking Union Medical College, Chinese Academy of Medical Sciences, Beijing; b Department of Endocrinology, Key Laboratory of Endocrinology, Fujian Provincial Hospital, Provincial Clinical College of Fujian Medical University, Fuzhou, China.

**Keywords:** GnRH pulse infusion therapy, gonadotropin, gonadotropin-releasing hormone, spermatogenesis

## Abstract

**Background:**

To compare the efficacies of gonadotropin-releasing hormone (GnRH) pulse subcutaneous infusion with combined human chorionic gonadotropin and human menopausal gonadotropin (HCG/HMG) intramuscular injection have been performed to treat male hypogonadotropic hypogonadism (HH) spermatogenesis.

**Methods:**

In total, 220 idiopathic/isolated HH patients were divided into the GnRH pulse therapy and HCG/HMG combined treatment groups (n = 103 and n = 117, respectively). The luteinizing hormone and follicle-stimulating hormone levels were monitored in the groups for the 1st week and monthly, as were the serum total testosterone level, testicular volume and spermatogenesis rate in monthly follow-up sessions.

**Results:**

In the GnRH group and HCG/HMG group, the testosterone level and testicular volume at the 6-month follow-up session were significantly higher than were those before treatment. There were 62 patients (62/117, 52.99%) in the GnRH group and 26 patients in the HCG/HMG (26/103, 25.24%) group who produced sperm following treatment. The GnRH group (6.2 ± 3.8 months) had a shorter sperm initial time than did the HCG/HMG group (10.9 ± 3.5 months). The testosterone levels in the GnRH and HCG/HMG groups were 9.8 ± 3.3 nmol/L and 14.8 ± 8.8 nmol/L, respectively.

**Conclusion:**

The GnRH pulse subcutaneous infusion successfully treated male patients with HH, leading to earlier sperm production than that in the HCG/HMG-treated patients. GnRH pulse subcutaneous infusion is a preferred method.

## 1 Introduction

Male hypogonadotropic hypogonadism (HH) is due to congenital or acquired factors from the hypothalamus, from the synthesis, transport, and secretion of pituitary gonadotropin-releasing hormone (GnRH), or from gonadotropin disorders. These acquired factors that cause HH lead to a series of clinical symptoms. According to the different etiologies of gonadotropin deficiency, HH is divided into congenital HH (CHH)^[1,2]^ and acquired HH (AHH).^[3]^ Defining the cause of AHH is relatively easy. Because intracranial tumors are the most common cause of AHH, magnetic resonance imaging (MRI) of the skull is an indispensable means of determining whether a patient potentially suffers from AHH. The clinical manifestations of AHH vary according to the time of onset. In contrast to that of AHH, the etiology of CHH is more complex. By studying the images of all kinds of endocrine tests and the sellar region showed that most CHH cases are idiopathic, which is the most common cause of idiopathic/isolated HH (IHH). The prevalence of IHH in men is approximately 1/10,000,^[4]^ and the proportion of those with male reproductive endocrine diseases in China has increased to 47.75%.^[5]^ HH of low gonadotropin is characterized by low gonadotropin and low sex hormone secretion, and the function of the testes is abnormal.^[6]^ Human chorionic gonadotropin (HCG) is commonly used in clinical practice as a substitute to luteinizing hormone (LH), and human menopausal gonadotropin (HMG) is used in the clinical setting as a replacement for follicle-stimulating hormone (FSH). The secretory level and spermatogenesis function of the testis can be recovered by its continuous stimulation with exogenous gonadotropin.^[7]^

The HH onset during puberty is characterized by an abnormal or delayed development of the external genitalia.^[8]^ Consequently, this abnormality causes infertility or sexual dysfunction. Based on the medical history of the patient, there are a number of tests that can be used to characterize sexual dysfunction during puberty. These tests include the following: physical examination, karyotype analysis, sex hormone level determination, the gonadotrophin-releasing hormone analog (GnRHa) stimulation test or the luteinizing-hormone-releasing hormone analog (LHRHa) stimulation test, bone age determination, hypothalamic pituitary MRI, if necessary, and the LH pulse analysis method for diagnosis and differential diagnosis.^[9]^ Upon reaching adulthood for these patients for whom HH was identified in puberty, the preferred treatment scheme, which involves preconditioning, ensures the success of spermatogenesis. HCG/HMG combined with intramuscular injection and GnRH pulsed infusion can treat HH and induce spermatogenesis.^[10]^ A vast amount of clinical data have confirmed that the application of HCG/HMG combined therapy can induce spermatogenesis in patients with HH,^[11]^ but the treatment cycle is long, and applications occur 2 to 3 times per week, which often leads to poor compliance by the patient. Belchetz et al^[12]^ clarified the physiologic mechanism of GnRH pulse secretion in 1978, and Hoffman and Crowley^[13]^ used gonadorelin (synthetic GnRH) pulse subcutaneous infusion for the 1st time in the treatment of patients with HH in 1982 and induced sperm production successfully. In China, Zhang and Jia^[14]^ and Sun et al^[15]^ reported in 2010 and 2011, respectively, successful study of pulsed subcutaneous infusion of GnRH for the treatment of HH spermatogenesis, but the sample size of these studies was relatively small.

Recently, a number of studies have shown that gonadotropin therapy for male patients with HH can effectively promote testicular volume (TV) increases and sperm production.^[16]^ However, the results of different studies on the effect and influencing factors of gonadotropin therapy for patients with HH are inconsistent.^[17]^ A meta-analysis suggested that gonadotropin treatment for HH produces a spermatogenic success rate of approximately 75%, but the spermatogenic success rate of before puberty-onset patients with HH was significantly lower than that of after puberty-onset patients with HH (68% [58–77%] compared with 84% [76–89%], *P* = .011).^[18]^ Another HH-based study (n = 74) found that the success rate of gonadotropin treatment was only 65%.^[19]^ The key to the success of HH treatment is the selection of an appropriate method combined with an appropriate dose of the drug. The main purpose of treatment is to maintain the function of the testicles, promote the development of secondary sex, improve the quality of life, and restore fertility. Testosterone preparation, gonadotropin, and pulse pump treatment with GnRH can be selected according to different needs. For male patients with reproductive needs, gonadotropin therapy is available. In this study, we analyzed the data of 220 patients with HH treated with spermatogenic treatment. We measured the number of days for the treatment of the 1st time sperm is found in the semen routine and the doses of spermatogenic drugs used for spermatogenesis and recorded any side effects. The main endpoint statistical method was compared with the difference between the number of spermatogenesis and testicular size before and after treatment, and clinical indicators were monitored. Furthermore, related factors of drug dosage and curative effect were analyzed. GnRH pulsatile subcutaneous infusion for the treatment of male patients with HH was compared with that for HCG/HMG treatment. Both groups were larger than those of previous studies and therefore afforded analysis of the optimal scheme for the clinical treatment of spermatogenesis.

## 2 Materials and methods

### 2.1 Materials

This randomized controlled trials study included 220 male patients with HH who were admitted to the department of endocrinology at Peking Union Medical College Hospital between January 2015 and December 2017. Random grouping was carried out according to the Consolidated Standards of Reporting Trials (CONSORT) process.

The clinical manifestation, history of cryptorchidism, history of drug use, and family history were recorded at the 1st visit. The physical examination included height, weight, age, vital signs, Tanner staging (breast, pubic hair), penis length, and TV (Prader testicle orchidometer). Laboratory examination included taking routine blood tests and measuring the blood biochemical index value, gonadotropin level (LH/FSH), total testosterone (TT) level, semen routine, LHRHa (Triptorelin) stimulation test, and pituitary/olfactory nerve MRI. This study was approved by the ethics committee of Peking Union Medical College Hospital.

The inclusion criteria of CHH diagnosis were made as follows: between the ages of 18 and 35 years when there is no secondary sexual characteristics or hypoplasia; a serum TT level <3.5 nmol/L, and LH and FSH levels in the normal ranges (inappropriately normal values); the functions of thyroid and adrenal and normal PRL; and Sellar region has no previous MRI space occupying lesions and surgery. Diagnosis of AHH was made if a patient met all of the following criteria: a male aged between 18 and 35 years without puberty development; a serum testosterone level <100 ng/dL (3.5 nmol/L) with low or normal levels of gonadotropins; a definite diagnosis of CHH. The exclusion criteria were as follows: single use of androgen replacement therapy; prior gonadotropin (HCG/HMG) spermatogenesis or GnRH pulse treatment for <6 months; and cryptorchidism.

### 2.2 Methods

#### 2.2.1 Grouping method

A total of 220 male patients with HH were voluntarily selected for GnRH pulse therapy or HCG/HMG therapy. Among them, 117 patients were treated with HGC/HMG (HCG/HMG group) and 103 patients received GnRH pulse therapy (GnRH group). Secondary endpoint: spermatogenesis was divided into 4 grades, namely, sperm densities >0 × 10^6^/mL, >5 × 10^6^/mL, >10 × 10^6^/mL, and > 15 × 10^6^/mL, to evaluate the time of treatment and changes in TV required by different levels of spermatogenesis. The patients were divided into 4 groups: GnRH-CHH, GnRH-AHH, HCG/HMG-CHH, and HCG/HMG-AHH. The success rate of each treatment was observed after 18 months. In each group, 10 patients with successful spermatogenesis were selected, and the relationship between TV and semen concentration was analyzed by analysis of variance. For those men who were married and had successful spermatism (i.e., men who have reached an adequate level of spermatogenesis to be fertile), the pregnancy and fertility of their wives were recorded.

#### 2.2.2 GnRH group treatment plan

A hormone pulse infusion pump (MicroPort Scientific Corporation, Shanghai, China) was used for gonadorelin pulse subcutaneous infusion (Fengyuan Pharmacy, Anhui, China; SFDA No. H10960064). The reference dose of GnRH is 100 to 500 μg/day. Each pulse infusion of gonadorelin (10 μg) was administered every 90 minutes (16 pulses/24 h). Based on the measured LH, FSH, and TT levels, the pulse infusion dose of gonadorelin was adjusted during the course of the treatment.

#### 2.2.3 HCG/HMG treatment scheme

Then, 5000 IU HCG (5000 IU/ampoule, Shanghai First Biochemical Pharmaceutical Co., Ltd., Shanghai, China; H31020865) and 75 IU HMG (75 IU/ampoule, Li Zhu Pharmaceutical Factory, Guangdong, China; SFDA No. H10940097) were used simultaneously. The reference dose of HMG was 75 to 150 IU intramuscular injection once or twice a week. HCG reference dose 5000 to 10,000 IU intramuscular injection once or twice a week. The drugs were mixed with 2 mL sterile water for injection, and then, a 5-mL syringe was used to draw the required volume of the drugs. This solution was injected into the gluteal muscles 2 times a week. The blood TT level 48 to 72 hours following the injection was measured, and the dose of HCG and HMG was adjusted according to the TT level and the production of spermatogenesis (the level of TT was maintained at 10–15 nmol/L).

#### 2.2.4 Follow-up method

After the treatment of spermatogenesis, follow-up of the GnRH group was performed in the 1st week and once per month thereafter until the 18th month (a total of 14 times). Medical checks were carried out once a month and at the 18th month for the HCG/HMG group (a total of 13 times). Changes to the secondary sexual characteristics (breast, penis, and TV) and any adverse reaction following intake of the medicine were recorded. The activity of the liver and kidney, blood routine, LH/FSH/TT levels, and semen were measured routinely.

#### 2.2.5 Hormone detection method

The LH, FSH, and TT levels were determined by double anti-sandwich chemiluminescence assays (commercial kits; Siemens Centaur, Bonn, Germany). There were 2.9% differences in LH and 2.4% in the batch, 3.9% in FSH and 4.5% in the batch, 5.6% in TT and 6.3% in the batch. Normal values for males were LH = 1.5 to 9.3 IU/L, FSH = 1.4 to 18.1 IU/L, and TT = 13.4 to 23.6 nmol/L. The detection limit for LH was 0.2 to 250 IU/L, for FSH was 0.1 to 200 IU/L, and for TT was 0.1 to 222 nmol/L.

The method of routine examination of semen involved 5 days of abstinence before collection of the semen. Semen was obtained by masturbation. WHO standards^[20]^ were used to analyze the semen volume, sperm density and sperm morphology. Sperm motility was divided into 3 groups: progressive motility (PR), nonprogressive motility (NP), and immotility (IM). The volume of the testicular testis (after natural descent or surgical fixation) was measured by the Prader testicle gauge assay, and the average volume of bilateral TV was also analyzed. The 2010 WHO reference range for sperm density is 15 million/mL.

#### 2.2.6 LHRHa (Triptorelin) stimulation test

Triptorelin stimulation test was performed at 8 am fasting state. An intramuscular injection of 100 mg triptorelin was used to determine the base value, and 60 minutes after the injection both the LH and FSH levels were determined. All patients completed the test at 1st visit.

### 2.3 Statistical analysis

The SPSS 19.0 software (SPSS Inc, Chicago, IL) was used for statistical analysis of the data. The measurement data, such as normal distribution, were described by the mean ± standard deviation 
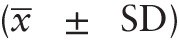
. If the distribution was skewed in accordance with the median, then the median (4th percentile) was used. Differences among the multiple follow-up data in the group were compared, and variance analysis of repeated measurements was used. Comparison of follow-up data differences from 2 groups was examined by the paired *t*-test, whereas comparison between groups was carried using the unpaired *t*-test, Chi-squared test and nonparameter test. Before and after treatment were compared using the paired-sample *t*-test. Analysis of the median treatment duration of spermatogenesis was carried out using the Kaplan–Meier approach. Prognostic factors were used to establish a Cox regression model to analyze the effects of spermatogenic therapy. All spermatogenic patients were used successfully to construct linear regression model analysis of the sperm early time. *P* < .05 suggested that the difference was statistically significant.

## 3 Results

### 3.1 Comparison of baseline clinical data and hormone levels in the GnRH and HCG/HMG groups

There was no significant difference in age, history of cryptorchidism, basal TV, and LH, FSH, and TT levels in the GnRH and HCG/HMG groups (Table 1). The patients with Kallmann's syndrome in the GnRH and HCG/HMG groups accounted for 50% and 55.7% of the patients in these groups, respectively (*P* = .526). All patients with HH were treated for more than 6 months and had not been treated with HCG or HMG previously. The median follow-up period for patients in the GnRH group was 18.4 (6.0–48.2) months, whereas for the HCG/HMG group, this value was 19.6 (6.0–48.8) months (*P* = .413).

**Table 1:**
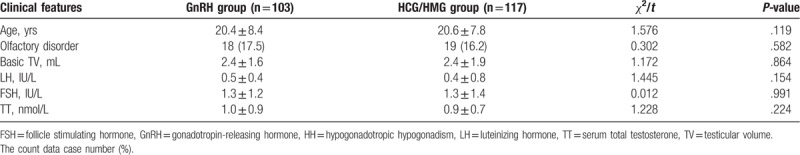
Baseline clinical data of the 2 groups of patients with HH.

### 3.2 The efficacy of GnRH pulse subcutaneous infusion for the treatment of HH

**Figure 1:**
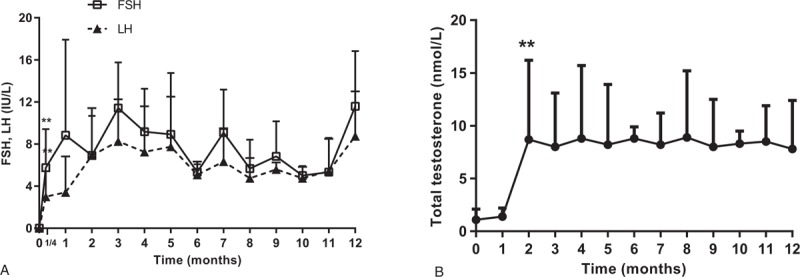
Changes in the luteinizing hormone (LH) and follicular-stimulating hormone (FSH) levels (A) and total testosterone (TT) levels (B) in the gonadotropin-releasing hormone group after treatment. Compared with before treatment: ∗∗*P* < .01 (1/4 month for 1 week).

After 1 week, LH (0.5 ± 0.4 vs 3.4 ± 2.4 IU/L, *P* < .01) and FSH (1.2 ± 1.2 vs 5.8 ± 3.8 IU/L, *P* < .01) increased significantly compared with the baseline levels (Fig. 1A). The TT levels after 3 and 6 months were 8.6 ± 7.2 and 7.9 ± 5.6 nmol/L, respectively, which were significantly higher than the those at baseline 1.0 ± 0.9 nmol/L, all *P* < .01 (Fig. 1B). During treatment, the level of LH was maintained between 3.4 ± 2.4 and 8.9 ± 4.1 IU/L. The FSH level was maintained between 5.8 ± 3.8 and 11.6 ± 5.2 IU/L, whereas the TT level was maintained between 7.9 ± 5.6 and 8.3 ± 6.5 nmol/L. The TV increased gradually. After 3 months of treatment, the TV increased from 2.3 ± 1.5 to 6.0 ± 2.5 mL (*P* = .001). At the last follow-up check, the TV was 8.1 ± 4.0 mL.

### 3.3 Therapeutic effect of HCG/HMG combined with intramuscular injection in the treatment of HH

The level of TT was 14.4 ± 8.0 nmol/L in the final follow-up and was significantly higher than that before treatment 0.8 ± 0.6 nmol/L (*P* < .01). The final follow-up level of TV was 7.6 ± 4.2 mL and was significantly higher than that before treatment 2.4 ± 2.1 mL (*P* < .01).

### 3.4 Comparison of spermatogenesis between the GnRH and HCG/HMG groups

**Figure 2:**
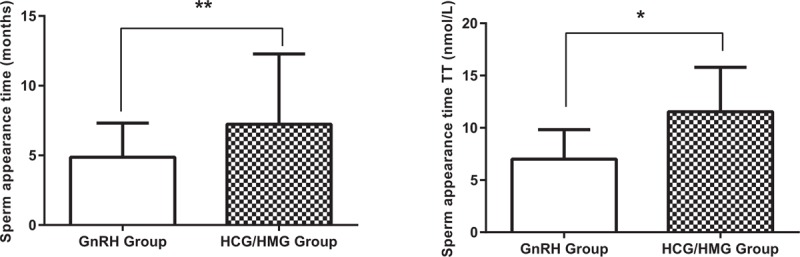
Comparison of sperm initial time (A) and sperm primary blood total testosterone level (B) in the gonadotropin-releasing hormone (GnRH) and human chorionic gonadotropin (HCG)/human menopausal gonadotropin (HMG) groups. TT: blood total testosterone; compared with the GnRH group. ∗*P* < .05, ∗∗*P* < .01.

Sperm was found in the semen of 62 patients (62/117, 52.99%) in the GnRH group, and the wife of one patient became pregnant naturally. In HCG/HMG group, there were 26 cases of spermatozoa (26/103, 25.24%, *P* = .032). The average sperm initial time of the GnRH group was 6.2 ± 3.8 months, whereas this value in the HCG/HMG group was 10.9 ± 3.5 months (*P* = .001; Fig. 2A). The average TV was 9.8 ± 3.3 mL in the GnRH group when the sperm 1st appeared, whereas this value in the HCG/HMG group was 8.1 ± 4.5 mL and *P* = .531. The last follow-up TVs in the GnRH and HCG/HMG groups were 10.3 ± 4.2 and 8.7 ± 4.5 mL, respectively (*P* = .619). The levels of primary TT in the GnRH and HCG/HMG groups were 8.3 ± 6.5 and 14.4 ± 8.0 nmol/L, respectively (*P* = .019; Fig. 2B). The levels of TT in the 2 groups had increased significantly after treatment. The TT level of 7.9 ± 5.3 nmol/L for the GnRH group was lower than that of the HCG/HMG group 14.1 ± 8.3 nmol/L (*P* < .01).

### 3.5 TV and influencing factors of spermatogenesis

**Figure 3:**
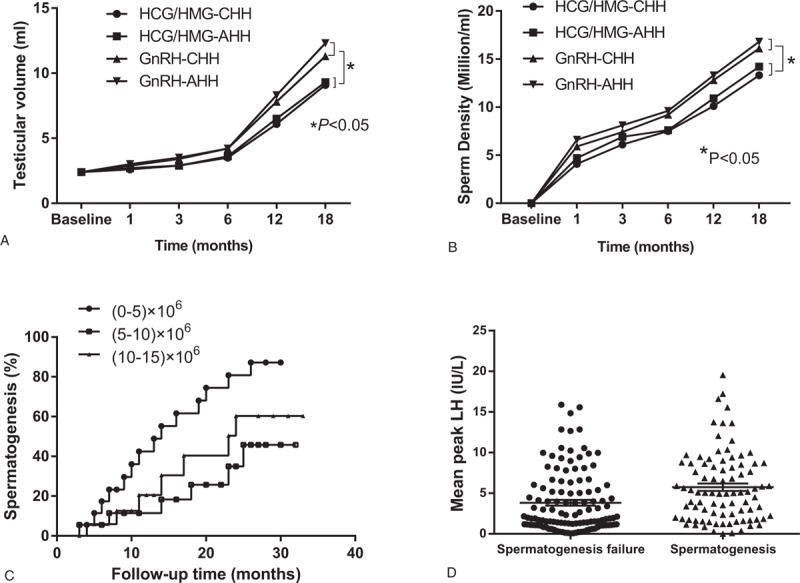
Influencing factors of spermatogenesis. The values in the chart are the median, mean and standard deviation. (A) The testicular volume of each group was observed 18 months after treatment. (B) Analysis of variance was used to analyze the relationship between treatment methods and sperm density. (C) All successful spermatogenesis patients (n = 88) required the median time to treat different sperm densities. A sperm density >0 × 10^6^/mL was reached after a median treatment period of 9 months. A sperm density >5 × 10^6^/mL was reached after a median treatment period of 13 months, whereas a sperm density >10 × 10^6^/mL was reached after a median treatment period of 18 months. (D) Comparison of the peak value of luteinizing hormone between the successful spermatogenesis group and the nonspermatogenic group. AHH = acquired hypogonadotropic hypogonadism, CHH = congenital hypogonadotropic hypogonadism, GnRH = gonadotropin-releasing hormone, HCG = human chorionic gonadotropin, HMG = human menopausal gonadotropin.

Secondary study endpoints: The patients were divided into 4 groups: GnRH-CHH, GnRH-AHH, HCG/HMG-CHH, and HCG/HMG-AHH. The TV of each group was observed 18 months after treatment. The TV was observed to increase faster after 6 months of regular treatment. Eighteen months later, the volumes were 11.3 ± 3.52 mL for the GnRH-CHH group, 12.2 ± 3.66 mL for the GnRH-AHH group, 9.5 ± 3.72 mL for the HCG/HMG-CHH group, and 9.1 ± 3.21 mL for the HCG/HMG-AHH group. There was no significant difference between the GnRH-CHH and GnRH-AHH groups or the HCG/HMG-CHH and HCG/HMG-AHH groups. A significant difference was observed between the GnRH treatment group (including GnRH-CHH, GnRH-AHH) and the HCG/HMG treatment group (including HCG/HMG-CHH, HCG/HMG-AHH), *P* < .05 (Fig. 3A). The success rates of spermatogenesis were 32/54 (59.3%) for the GnRH-CHH group, 30/49 (61.2%) for the GnRH-AHH group, 14/62 (22.6%) for the HCG/HMG-CHH group, and 12/55 (21.8%) for the HCG/HMG-AHH group. In each group, 10 patients who had successful spermatogenesis were selected, and the relationship between the treatment method and sperm density was examined by variance analysis. The sperm density of the 4 groups were (10.28 ± 5.19) × 10^6^ for the GnRH-CHH group, (11.76 ± 6.51) × 10^6^ for the GnRH-AHH group, (8.62 ± 4.57) × 10^6^ for the HCG/HMG-CHH group, and (8.75 ± 4.61) × 10^6^ for the HCG/HMG-AHH group. The sperm density of the GnRH pump treatment group was clearly better than that of the HCG/HMG treatment group (Fig. 3B). In addition, after 18 months, the sperm motility A level (progressive motility) was more than 25%, and the B level (nonprogressive motility) ≥50% in all patients with spermatogenesis, for whom the sperm shape was observed to be normal. Eighteen months after treatment 7 of the spouses who had partners in the GnRH treatment group were pregnant, whereas 2 women from those in the HCG/HMG group were pregnant.

The results of Kaplan–Meier analysis showed that the success of the spermatogenesis patients (n = 88) had a median treatment time of 9 months (95% confidence interval [CI]: 5.4–12.6) and an average TV of 7.3 ± 3.1 mL in the patients’ spermatogenesis (sperm density >0/mL). The median time for the sperm density to reach >5 × 10^6^/mL was 13 months (95% CI: 11.2–14.8), and the mean volume of the testis was 8.9 ± 3.2 mL. The median time for the sperm density to reach >10 × 10^6^/mL was 18 months (95% CI: 15.3–20.7), and the mean volume of the testis was 9.6 ± 3.3 mL. The sperm density did not reach >15 × 10^6^/mL (Fig. 3C). In the triptorelin excitation test, we compared the level of LH 60 minutes after injection of GnRHa between the successful (n = 88) and unsuccessful spermatism patients (n = 112). The LH values were higher in the successful group (2.3 [0.7, 7.0] vs 1.5 [0.4, 2.8] IU/L, *P* = .010) (Fig. 3D).

## 4 Discussion

The type of treatment for male HH can be chosen according to the needs of the patient. Patients who have no intention of having a family can take an androgen supplement to promote and maintain male secondary sex and improve metabolism and bone density. Those patients seeking to have a family need to consider GnRH pulse infusion and HCG/HMG treatment combined with intramuscular injections in the treatment of spermatogenesis dysfunction. In this study, 220 male patients with HH were treated with HCG/HMG and GnRH pulse infusion. The TT level in the serum of patients reached normal levels, and the volume of the testicles was clearly observed to increase. As a result, 56% of the patients successfully produce spermatozoon. Patients with HH can be treated effectively by gonadotropin therapy to alleviate spermatogenesis dysfunction.

The results of this study showed that LH and FSH were significantly increased after 1 week of GnRH pulse therapy. Varimo et al^[4]^ showed that the level of LH and FSH increased significantly after 1 to 2 weeks of GnRH pulse therapy, which is consistent with the results herein. Sun and Dou^[5]^ and our research results showed that during long-term GnRH pulse treatment, LH and FSH levels can be maintained in the physiologic range of normal adult men, reflecting that GnRH pulse infusion treatment of HH is consistent with physiologic conditions.

The TV of the patients in group GnRH and group HCG/HMG increased significantly, but the TV of the 2 groups did not reach 15 to 20 mL, which is within the normal range of adult TV.^[9]^ This observation may not be related to the length of the treatment time. Longer GnRH and HCG/HMG treatments may further increase the volume of the testicles; however, a lower volume may be associated with a developmental disorder of the testes caused by HH.^[16]^ In the 2 groups, the initial sperm TV was ∼9 mL, which is consistent with the primary TV of 4 to 12 mL in normal males.^[21]^ Although there was no difference in TV between the 2 groups, the spermatogenesis time in the GnRH group was shorter, suggesting that attaining active spermatogenesis was faster in the GnRH pulse therapy group.

The rates of spermatogenesis in the GnRH HCG/HMG groups were 52.99% (62/117) and 25.24% (26/103), respectively. This result suggests that the rate of spermatogenesis is higher when GnRH pulse therapy is used in a short-term treatment program. With longer treatment periods, the final sperm production rate in the GnRH and HCG/HMG groups is not different. The study by Liu et al^[22]^ showed that after 2 years of follow-up, the rate of GnRH pulsed treatment of spermatogenesis was equivalent to that of the combined treatment of HCG/HMG. However, in this study, only 5 patients with HH received GnRH pulse therapy, and this small population likely affects the reliability of the conclusions drawn. We still need to lengthen the observation period to determine the rate of final sperm production in the 2 groups.

It is generally believed that the volume of the testicles before treatment is one of the major factors affecting the prognosis of patients with HH. The TV (>4 mL) before treatment and the effect of treatment for adolescent development is improved to a certain degree. Rohayem et al^[23]^ studied 60 male patient with HH with ages ranging between 14 and 22 years. They found that HCG/rFSH replacement therapy increased TV and that the sperm concentration was normal. The replacement of hCG/rFSH in adolescence successfully induced testicular growth and spermatogenesis, which was independent of previous testosterone replacement and could improve quality of life. Zacharin et al^[24]^ studied 19 male hypogonadism patients. HCG treatment alone or in combination with recombinant follicle stimulating hormone (rFSH) for 6 to 9 months has been carried out. This study evaluated the effect of rFSH on the treatment of chorionic gonadotropin (HCG) in young male adolescents with HH. The median time for successful spermatogenesis was 9 months. The rFSH combined with HCG in treating puberty or young male HH promotes normal growth of testis and promotes spermatogenesis. Most men need treatment for at least 2 years to maximize TV and sperm production. This regimen only applies to patients with normal pituitary function.

The sperm initial times of the GnRH and HCG/HMG groups were 6.2 and 10.9 months, respectively. The results of the Büchter et al^[25]^ study showed that the mean time of HH sperm initial onset was 6 months when GnRH pulse infusion treatment was applied, which is similar to our results. Our results also showed that the initial sperm TT level was lower in the GnRH group than in normal adult males, whereas the HCG/HMG group reached normal adult levels. The possible reasons are as follows: 1st, although the level of LH and FSH was stable at normal levels, there is the possibility that testicular Leydig cells were dysfunctional, or patients did not experience physiologic mini-puberty and the number of Leydig cells decreased.^[26]^ Moreover, in the GnRH group, although the LH and FSH levels were stable at normal levels, Leydig cells decreased due to possible testicular Leydig cell dysfunction, or the absence of physiologic mini-puberty. In the HCG/HMG group, after an intramuscular injection of HCG, TT levels fluctuated bimodally, with the 1st and 2nd peaks (the highest peaks) occurring at 2 to 7 and 48 to 72 hours. The TT level of the HCG/HGM group was measured 48 to 72 hours after injection, which just reflects the peak of the TT level. We postulate that the actual average TT level of the HCG/HMG group may be lower than the test result presented. Second, GnRH pulse therapy can promote the stability of pulse type pituitary LH secretion, and the level of TT is kept stable for a long period. However, the level of TT was shown to fluctuate significantly by HCG by Żarski et al.^[27]^ These differences make the testosterone levels in the testicles of the GnRH group more stable than those of the HCG/HMG group, thereby promoting sperm production earlier. Third, human spermatogenic cells and mature spermatozoa express the GnRH receptor,^[28]^ and there is GnRH in semen. Therefore, there may be GnRH autocrine and paracrine roles in the testis that are involved in regulating the production and maturation of spermatozoa. Thus, it is plausible that GnRH pulse therapy may promote directly spermatogenesis and maturation of the testis.

For patients with HH, GnRH pulse pump therapy and traditional gonadotropin therapy can promote the development of gonadal, sex hormone synthesis, and reproductive cell maturation. Continuous pulse type is given to GnRH analogs that make the pituitary produce a normal gonadotropin pulse, which induces the development of the gonadal glands, synthetic hormones, and the maturation of the germ cells. Spermatogenesis is regulated by gonadotropin-regulated testicular RNA helicase (GRTH/DDX25). GRTH/DDX25 is a testicular-specific DEAD box protein family of RNA helicase, which exists in testis and germ cells. The GRTH gene is regulated by the rise of chorionic gonadotropin (HCG), and it shows a specific expression in the process of germ cell development.^[29]^ The protein products encoded by the GRTH gene have ATPase activity and ATP-dependent bi-directional RNA helicase activity, and participate in the regulation of RNA translation, transcription, and splicing. The gene is mainly expressed in Sertoli cells, spermatocyte, and spermatozoa, and is expressed sequentially and specifically during spermatogenesis. Its products can promote protein translation in vitro, suggesting that the GRTH gene may be closely related to the regulation of gene expression during spermatogenesis.^[30]^ GRTH can also prevent the apoptosis of sperm cells. Research shows that GRTH, as a component of mRNP (messenger ribonuclear protein), promotes NF-kappaB function by negative regulation of tumor necrosis factor receptor 1 and caspase pathway to prevent the apoptosis of spermatocyte.^[31]^

This study is a randomized controlled trials analysis, and it has some limitations. First, further prospective studies are required to examine the history of testosterone or gonadotropin therapy, and the effect this therapy has during the initial stages of spermatogenesis and on the efficacy of spermatogenesis. In addition, mutations in at least 20 genes can cause HH. Different gene mutations may be related to spermatogenic consequences. The correlation between genotype and spermatogenic efficacy is not understood and needs further characterization. However, we believe that this study will benefit a wide audience ranging from endocrinology clinicians to male patients with HH.

## 5 Conclusion

Our study suggested that GnRH pulse infusion therapy simulates the physiologic secretion of the human GnRH pulse, which is more consistent with the physiologic state. Compared with the combined treatment of HCG/HMG, GnRH pulse subcutaneous infusion can promote spermatogenesis faster. Therefore, GnRH pulse subcutaneous infusion is an optimal choice for the treatment of spermatogenesis in patients with HH.

## Acknowledgment

The authors thank Dr Zhao-Xiang Liu and Qi-bing Huang (PUMCH, CAMS, Beijing, MD) for collecting a part of male patients with HH.

## Author contributions

**Data curation:** Jianli Lin, Jiang-Feng Mao, Xi Wang, Wan-Lu Ma, Ming Hao.

**Formal analysis:** Jianli Lin, Jiang-Feng Mao, Ming Hao.

**Funding acquisition:** Xue-Yan Wu.

**Investigation:** Jianli Lin, Xi Wang, Wan-Lu Ma.

**Project administration:** Jianli Lin, Xue-Yan Wu.

**Supervision:** Xue-Yan Wu.

**Writing – original draft:** Jianli Lin.

**Writing – review & editing:** Jianli Lin, Jiang-Feng Mao, Xi Wang, Ming Hao, Xue-Yan Wu.
